# Complete Common Bile Duct Injury after Laparoscopic Cholecystectomy in Situs Inversus Totalis: A Case Report, Review of the Literature and Illustrative Case Video

**DOI:** 10.1016/j.ijscr.2024.109265

**Published:** 2024-01-17

**Authors:** Bahaa I. Aburayya, Layan R. Obeidat, Farah I. Kitana, Omar Al Khatib, Saleh Romman, Osama H. Hamed

**Affiliations:** a Faculty of Medicine, Jordan University of Science and Technology, Irbid 22110, Jordan; b Specialty Hospital, Jaber Ibn Hayyan St, Amman, Jordan; c Arab Medical Center, Jabal Amman, 5th Circle, Amman 11181, Jordan

**Keywords:** Case Report, Common Bile Duct Injury, Laparoscopic Cholecystectomy, Situs Inversus Totalis, Hepaticojejunostomy

## Abstract

**Introduction and importance:**

Situs Inversus Totalis (SIT) is a rare condition characterized by the transposition of internal organs. Given the anatomical variations in SIT, surgeons must exercise extreme caution when performing laparoscopic cholecystectomy to avoid iatrogenic bile duct injury. Despite the high difficulty index associated with laparoscopic cholecystectomy in SIT, there is only one case report of common bile duct (CBD) injury in the English-language literature.

**Case presentation:**

A 41-year-old female patient, known to have Kartagener syndrome, underwent laparoscopic cholecystectomy for acute cholecystitis and was discharged home on post-operative day one. However, on post-operative day five, the patient presented to the emergency room with abdominal pain, fever, and jaundice. Laboratory tests revealed leukocytosis and hyperbilirubinemia. Radiological images revealed complete occlusion of the CBD. A delayed approach was chosen, and six weeks after her initial operation, a hepaticojejunostomy was performed.

**Clinical discussion:**

Laparoscopic cholecystectomy is the standard operative procedure for gallbladder disease. The unique anatomy of SIT increases the risk of CBD injury during laparoscopic cholecystectomy. Surgeons are required to perform a mirror-image procedure and adhere to the basic principles of safe laparoscopic cholecystectomy in SIT. This is only the second reported case of CBD injury in SIT patients.

**Conclusion:**

Laparoscopic cholecystectomy in SIT presents a significant challenge. In patients with SIT, preventing CBD injury is the best approach, and referral to an experienced hepato-pancreato-biliary (HPB) surgeon is recommended. A delayed approach to CBD injuries in SIT allows thorough planning and understanding of the complex anatomical variations in these patients.

## Highlights

• Situs Inversus Totalis poses a challenge for practicing surgeons during laparoscopic cholecystectomy due to anatomical variations.
• The risk of iatrogenic common bile duct injury in SIT increases during acute surgical conditions such as acute cholecystitis.
• Radiological investigations should be implemented if common bile duct injury is suspected
• Prevention of CBD injuries is considered the best approach and referral to an experienced Hepato-pancreato-biliary surgeon is recommended.
• A delayed approach to repairing common bile duct injuries in patients with Situs Inversus Totalis via hepaticojejunostomy is advised.

## 1 Introduction

Situs Inversus Totalis (SIT) is a rare autosomal recessive condition characterized by the transposition of the thoracic and abdominal viscera, resulting in a mirror image of the normal anatomy [1]. The incidence of SIT has been reported to vary widely, ranging from 1:5000 to 1:20,000 [1,2]. Patients with SIT pose a major challenge for surgeons due to their significant anatomical variations [3]. The difficulty index increases during acute surgical conditions since the surgeon may not have the luxury of time to prepare for such a challenging situation, which makes the diagnosis and management of acute cholecystitis difficult in this group of patients [3]. Nevertheless, several previous case reports have successfully described the performance of laparoscopic cholecystectomy in these patients, with no reported common bile duct (CBD) injury [3]. In a recent “Letter to the Editor,” Usta et al. reported the first case of CBD injury in a 61-year-old man with SIT after a laparoscopic converted to open cholecystectomy [4].

In accordance with the SCARE criteria [5], we present the second case of CBD injury following laparoscopic cholecystectomy in a patient with SIT. This case report aims to discuss the various details involved in managing such a challenging situation and describes the chosen delayed approach for successfully repairing this injury.

## 2 Case Presentation

**Fig. 1:**
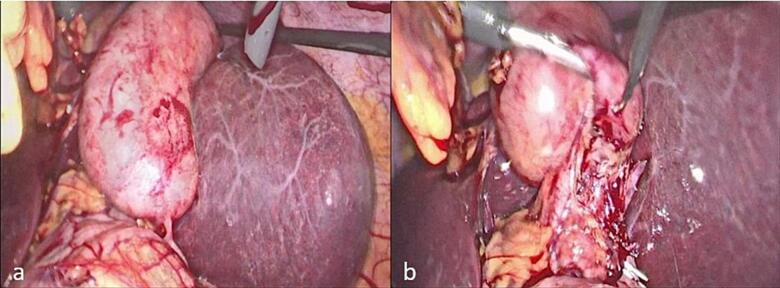
Intraoperative view during laparoscopic cholecystectomy. (a) Severely inflamed gallbladder with a thickened wall, (b) hands of the surgeon are crossed.

A 41-year-old female patient, known to have Kartagener syndrome (SIT, chronic sinusitis, and bronchiectasis) [6], as well as asthma (on Advair 250/50), and a history of two C-sections (in 2003 and 2005), presented to the emergency department with left upper quadrant abdominal pain associated with fever, nausea, and vomiting. An ultrasound revealed acute cholecystitis, and the patient was taken to the operating room for laparoscopic cholecystectomy, performed by a practicing general surgeon. In the operating room, after gaining access to the abdomen, it was discovered that the patient had a severely inflamed gallbladder with a thickened wall (Fig. 1a). The surgeon attempted to provide a mirror image approach in the trocar position but was unsuccessful due to his hand position and function, as shown in the video of the operation (please see the video). The surgeon's hands were crossed, with the right-hand dissecting and the left retracting (Fig. 1b). The gallbladder was successfully dissected from the liver bed, and the surgery was completed. The patient was discharged home on post-operative day one after the removal of the abdominal drain, which contained serous non-biliary output.

On post-operative day five, the patient presented to the emergency room with abdominal pain, fever, and jaundice. Laboratory tests revealed an elevated white blood cell count (15,000/mm^3^) and direct hyperbilirubinemia (direct bilirubin 4.3 mg/dL, total bilirubin 5.4 mg/dL).

**Fig. 2:**
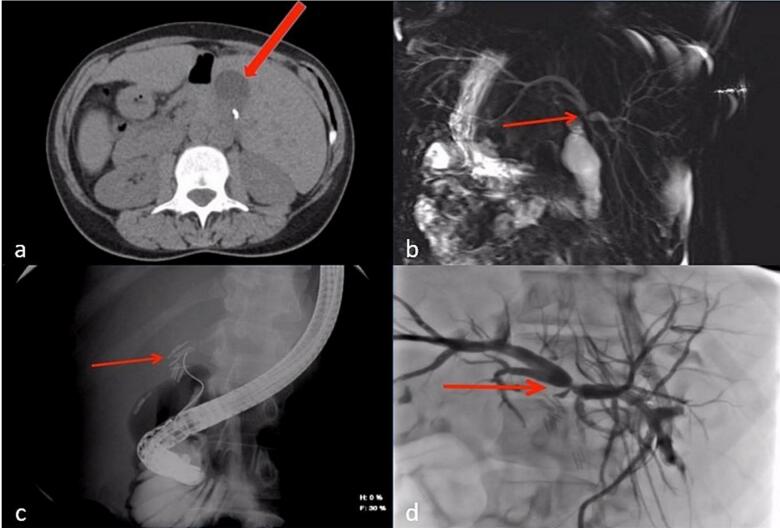
Series of images after the suspected biliary injury. (a) Perihepatic fluid collection on non-contrast CT scan, (b) MRCP showing complete occlusion of the CHD just below the bifurcation (Bismuth-Strasberg Type E3 bile duct injury), (c) ERCP showing complete occlusion of the CHD just below the bifurcation with multiple clips, (d) PTC showing dilation of the biliary tree and occlusion of the CHD (Bismuth-Strasberg Type E3 bile duct injury).
• Magnetic resonance cholangiopancreatography (MRCP) was obtained, revealing complete occlusion of the common hepatic duct (CHD) just below the bifurcation of the right and left hepatic ducts (Bismuth-Strasberg Type E3 bile duct injury) [7] (Fig. 2b).
• Endoscopic retrograde cholangiopancreatography (ERCP) was attempted, though cannulating the duct was challenging due to her abnormal anatomy. It showed complete occlusion of the CBD and CHD with multiple clips (Fig. 2c).
• Percutaneous transhepatic cholangiography (PTC) was also performed, revealing biliary tree dilatation with complete occlusion of the CHD just below the bifurcation (Bismuth-Strasberg Type E3 bile duct injury) [7] with multiple large clips at the occlusion site (Fig. 2d).
• A contrast-enhanced CT scan confirmed the appropriate positioning of the PTC catheters and identified abnormal arterial anatomy, including a replaced right hepatic artery (RHA) originating from the superior mesenteric artery (SMA) (Fig. 3a), a replaced left hepatic artery (LHA) originating from the gastroduodenal artery (Fig. 3b), and an accessory left hepatic artery originating from the left gastric artery (Fig. 3c).

A series of images were obtained as follows:

• A non-contrast CT scan of the abdomen revealed a small perihepatic fluid collection (Fig. 2a).

**Fig. 3:**
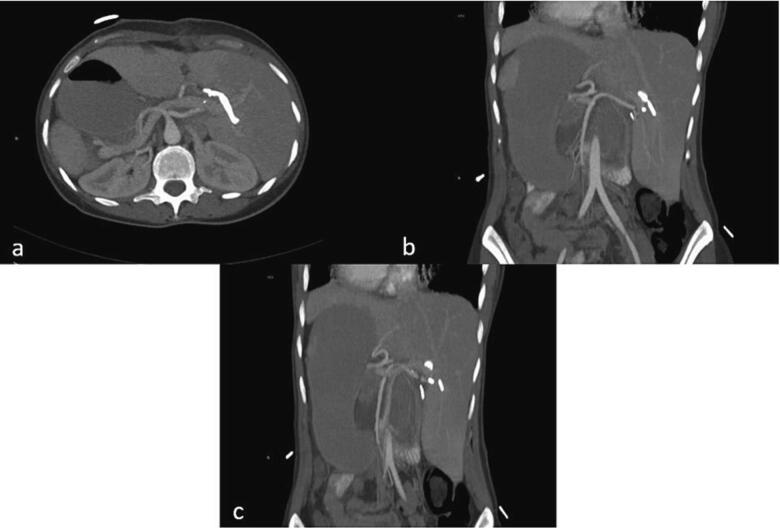
Contrast-enhanced CT scan showing abnormal arterial anatomy. (a) Replaced right hepatic artery, (b) replaced left hepatic artery, (c) accessory left hepatic artery.

**Fig. 4:**
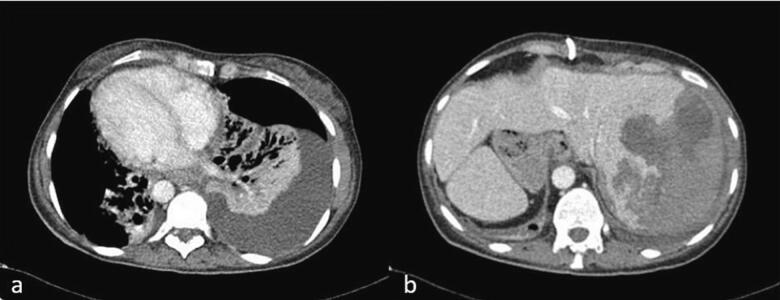
CT scan showing hospital course complications. (a) Left-sided pneumonia, (b) large subcapsular hematoma in the liver from PTC.

Her hospital course was complicated with pneumonia on top of her pre-existing bronchiectasis (Fig. 4a) and a large subcapsular hematoma of the liver resulting from the PTC procedure (Fig. 4b), requiring multiple blood transfusions and IV antibiotics for the pneumonia. Four weeks later, her PTC stents were upsized from 10 Fr to 12 Fr. A follow-up CT scan revealed a resolution of the subcapsular hematoma, and the decision was made to proceed with a hepaticojejunostomy six weeks after her initial operation.

## 3 Operative Procedure

**Fig. 5:**
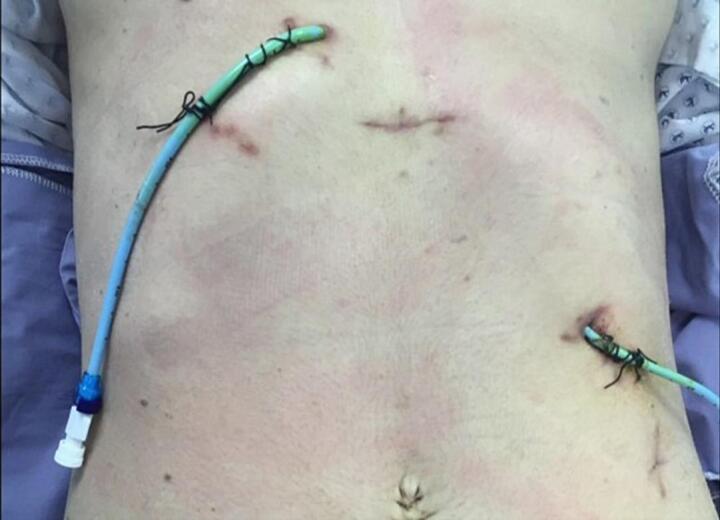
Sites of the PTC catheters during the operative repair.

**Fig. 6:**
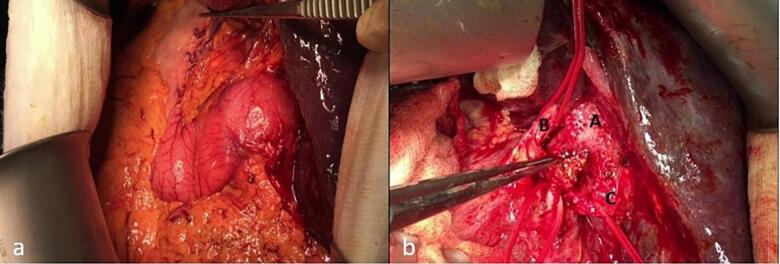
Intraoperative view during hepaticojejunostomy. (a) Orientation of the duodenum with the liver in the left upper quadrant, (b) CHD with multiple clips (letter A), replaced right hepatic artery (letter B), and replaced left hepatic artery (letter C).

**Fig. 7:**
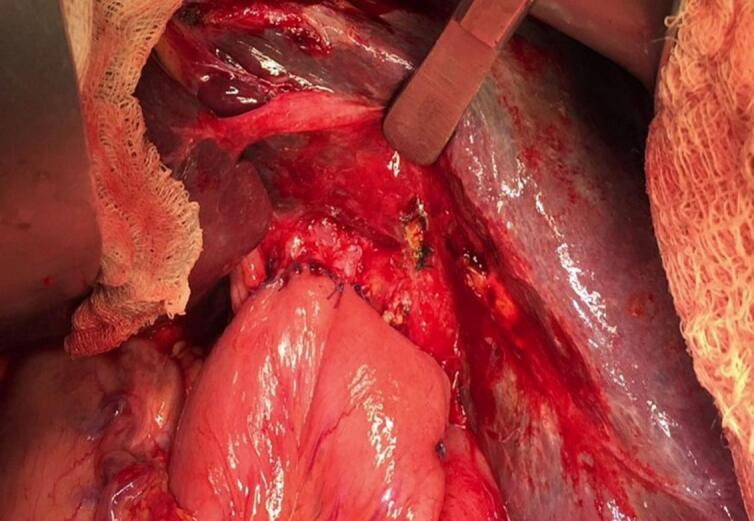
Final result after standard hepaticojejunostomy.

**Fig. 8:**
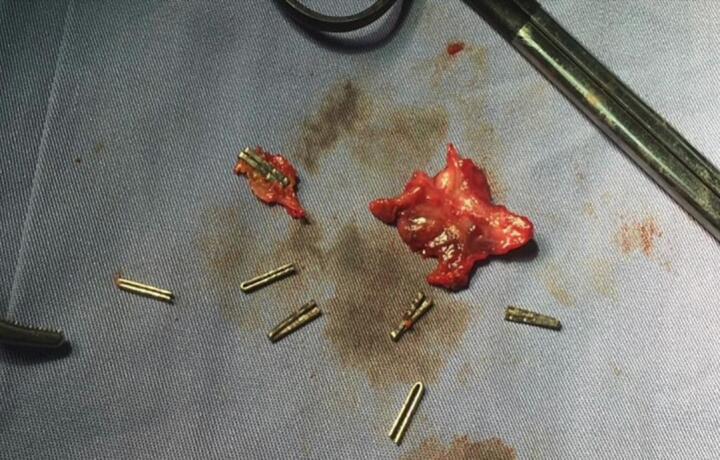
The specimen after careful dissection with a total of 9 clips.

Fig. 5 shows an image displaying the sites of the PTC catheters. Intraoperatively, we identified the orientation of the duodenum, with the liver in the left upper quadrant and the stomach in the right upper quadrant (Fig. 6a). After meticulous dissection of the porta hepatis, we identified a replaced left hepatic artery, a replaced right hepatic artery, and the CBD with multiple clips just below the CHD bifurcation (Fig. 6b). The CHD was transected just above the site of clip occlusion. The PTC catheters were then exteriorized from the openings, and the thick, hard PTC catheters were replaced with soft biliary drains over the guidewire. We proceeded with a standard Roux-en-Y hepaticojejunostomy performed by a practicing hepato-pancreato-biliary (HPB) surgeon. The final result is depicted in Fig. 7. After carefully dissecting the specimen, we identified a total of nine clips on the specimen (Fig. 8). The patient recovered well from the surgery without complications. The soft PTC catheters were removed after 2 weeks, and the patient had normal liver function with no issues related to the surgery during the 4-year follow-up period.

Please refer to the accompanying video presentation for more detailed information about the case.

## 4 Discussion

Laparoscopic cholecystectomy is the standard operative procedure for gallbladder disease and is the most commonly performed general surgery procedure worldwide [8]. Over the last three decades, extensive efforts have been made to optimize outcomes and minimize complications, particularly iatrogenic bile duct injuries (BDI) that continue to be the primary concern after gallbladder surgery [7]. Perhaps one of the most widely accepted approaches is the identification of the Critical View of Safety (CVS), as described by Strasberg et al. [9] and the selective use of intraoperative cholangiogram [10].

The reported incidence of iatrogenic BDI in patients undergoing laparoscopic cholecystectomy ranges between 0.07 and 1.1 % in the literature [11,12]. While there is no evidence to suggest that SIT increases the risk of BDI following laparoscopic cholecystectomy [13], the anatomical variation associated with SIT is a potential risk factor that may contribute to a higher incidence of BDI after the procedure [14,15]. Furthermore, several other factors may contribute to an increased risk of BDI in patients with SIT:

1. A delay in the diagnosis and management of gallbladder disease can occur if the patient's SIT status is unknown beforehand [3]. This delay may result from the atypical presentation of LUQ pain, which can sometimes be confused with pancreatitis [4].
2. Currently, there is no standardized technique for the optimal management of gallbladder disease in patients with SIT [3]. However, some techniques have been proposed to reduce the risk of BDI after laparoscopic cholecystectomy, which can be employed [14].
3. The reversed anatomy in patients with SIT presents a challenge for right-handed surgeons, while left-handed surgeons may have an advantage.

In patients with SIT, laparoscopic surgery represents a significant challenge for the operating surgeon and the operating room team in various aspects. The team should be ready to perform a mirror-image procedure at all levels, considering the target organ's new mirror-image position within the abdominal or thoracic cavity; this involves adjusting the positions of the surgeon, assistants, and the monitor, as well as repositioning trocars and instruments, taking into account the anatomical variations common in patients with SIT [16].

The surgeon must also reorient his surgical skills to the LUQ during cholecystectomy. All of these factors contribute to the complexity of the case, requiring the surgeon to overcome the influence of habitual thinking in his operative techniques for commonly performed laparoscopic procedures to avoid complications and achieve successful outcomes [16]. The complexity is further amplified during acute surgical conditions because the surgeon will not have the luxury of time to mentally prepare for such challenging situations that demand extensive surgical modifications.

The first successful laparoscopic cholecystectomy in a patient with SIT was performed in 1991 by Campos and Sipes [17]. Since then, more than 90 cases of successful laparoscopic cholecystectomy in patients with SIT have been reported [3]. Additionally, multiple reports have demonstrated the successful utilization of laparoscopic surgery in patients with SIT for various surgical operations [[18], [19], [20]].

**Fig. 9:**
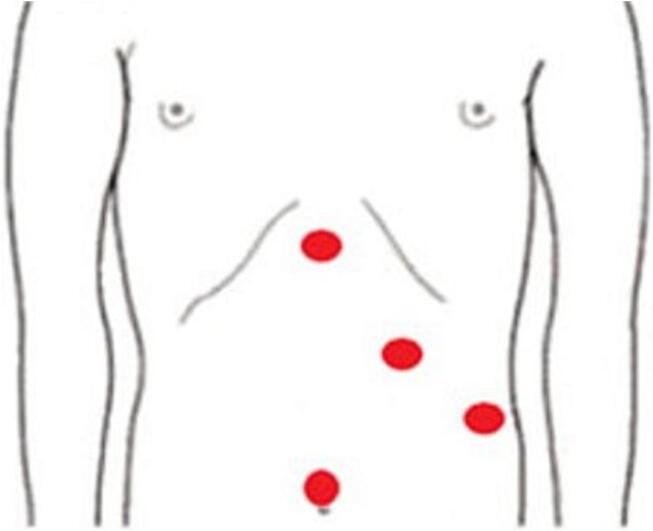
Operation field photograph showing ports placed in the mirror positions.

In a recent systematic review of laparoscopic cholecystectomy in SIT, Chaouch et al. identified 93 cases of successfully performed procedures with no reported iatrogenic BDI or significant complications [3], indicating the safety of the procedure despite its challenging aspect. A detailed analysis of the 93 reported cases revealed that the most commonly utilized technique was the 4-port “American Mirror Technique” (Fig. 9), with the surgeon and his assistant positioned on the patient's right side and the monitor on the left. Most surgeons were right-handed, and the median reported operative time was 74 min (range: 30–240 min), and only one case of acute cholecystitis required conversion to open due to difficulty in identifying the cystic duct. In another recent literature review by Alkhlaiwy et al., chronic cholecystitis was found to be the main indication for surgery in the majority of SIT cases, with acute cholecystitis reported in only 11 out of the 92 cases [21]. The reported cases in the literature highlight the extra caution and attention given to preparation and planning in both elective and emergency settings. However, this was not the case with our patient, who was immediately rushed from the emergency room to the operating room after the diagnosis of acute cholecystitis.

To avoid BDI, it is essential to adhere to the basic principles of safe laparoscopic cholecystectomy, including the identification of the Critical View of Safety (CVS) (which was not identified in our case), the selective use of intraoperative cholangiogram, and the low threshold of conversion to open surgery in cases of poor progression during the procedure [3,21]. Some reports have suggested referring such patients to left-handed surgeons or a tertiary referral to an experienced HPB center if available to limit complications [21,22].

The management of BDI depends on the timing, type, and extent of the BDI. Various approaches can be employed, including endoscopic, surgical, and radiological [14]. Surgeons are advised to begin by utilizing all available radiological modalities, including ultrasound, CT, MRI, ERCP, and MRCP (the gold standard in BDI classification [14,23]), to identify the type of BDI and plan the appropriate management [24]. If imaging reveals a bile leak or fluid collection, immediate placement of a drain under radiological guidance is recommended [14].

Our management began with a careful review of the videos from the original laparoscopic cholecystectomy surgery and suspected CBD injury. We implemented a standard stepwise approach for CBD injury after laparoscopic cholecystectomy in terms of workup, diagnosis, and management. Given the complexity of the situation, we utilized all available imaging modalities, including CT scans, MRCP, ERCP, and PTC, to accurately identify the type of injury and facilitate the management of this complication. Due to the complexity of the case and multiple associated co-morbidities, a delayed approach was chosen for this patient. Ultimately, the CBD injury was successfully managed with a standard Roux-en-Y hepaticojejunostomy [25].

This case report is the second reported case of complete CBD injury after laparoscopic cholecystectomy in a patient with SIT, despite the challenging nature of this procedure. Perhaps the very low reported rate of BDI is due to the extra care and attention the surgeons provide in this scenario or the tendency to underreport complications and mishaps in general [4].

## 5 Conclusion

Despite the numerous case reports documenting successful laparoscopic cholecystectomy in patients with SIT, it is crucial to remain aware that iatrogenic bile duct injury remains a significant concern during gallbladder surgery. All efforts should be made to prevent such a complication. We hope that our case report will shed some light on this issue, and we trust that young surgeons will learn from these experiences and approach such challenging scenarios with the utmost seriousness in the future.

The following are the supplementary data related to this article.

Video 1 Case presentation. Video 1

Video 2 Cholecystectomy. Video 2

## Informed consent

Written informed consent was obtained from the patient for publication of this case report and accompanying images. A copy of the written consent is available for review by the Editor-in-Chief of this journal on request.

## Ethical approval

Case reports are exempt from ethical approval in our institution.

## Funding

This research did not receive any specific grant from funding agencies in the public, commercial, or not-for-profit sectors.

## Guarantor

The corresponding author, Osama H. Hamed.

## Research registration number

Not applicable.

## CRediT authorship contribution statement

All authors were involved in the conceptualization, data curation, investigation, methodology, project administration, resources, software, validation, visualization and writing the original draft. Osama H. Hamed and Bahaa I. Aburayya were involved in reviewing and editing the final manuscript.

## Declaration of competing interest

The authors declare that they have no conflicts of interest.
